# Dual role of lactate in human health and disease

**DOI:** 10.3389/fphys.2025.1621358

**Published:** 2025-08-01

**Authors:** Sudhir Kumar, Neha Sahu, Talha Jawaid, Hanish Singh Jayasingh Chellammal, Prabhat Upadhyay

**Affiliations:** ^1^ Department of Microbiology and Immunology, School of Medicine, Emory University, Atlanta, GA, United States; ^2^ Department of Botany, University of Lucknow, Lucknow, Uttar Pradesh, India; ^3^ College of Medicine, Imam Mohammad Ibn Saud Islamic University (IMSIU), Riyadh, Saudi Arabia; ^4^ Department of Pharmacology and Life Sciences, Faculty of Pharmacy, Universiti Teknologi MARA (UiTM), Puncak Alam, Selangor, Malaysia; ^5^ Vincent Centre for Reproductive Biology, Department of Obstetrics and Gynecology, Massachusetts General Hospital, Harvard Medical School Boston, Boston, MA, United States

**Keywords:** lactate, lactate dehydrogenase, cancer, metabolic disorders, gut microbiota

## Abstract

Lactate, traditionally seen as a byproduct of anaerobic metabolism, has gained attention for its dual role in human health. While it is associated with muscle fatigue, lactate also plays a crucial role in various physiological and pathological processes. This review explores lactate’s dual nature as both beneficial and detrimental. Under normal physiological conditions, lactate is an essential energy substrate, involved in the Cori cycle, where it is converted back to glucose in the liver. However, excessive lactate accumulation is linked to health issues, including cancer, metabolic disorders, and neurological diseases. The Warburg effect in cancer, characterized by increased lactate production even in oxygen-rich environments, promotes tumor progression and therapy resistance. In diseases like malaria and ischemic stroke, high lactate levels contribute to tissue damage and metabolic disturbances. Recent research also highlights lactate’s beneficial roles, including regulation of immune responses, enhanced exercise performance, and neuronal signaling. Furthermore, gut microbiota significantly impacts lactate metabolism, where beneficial bacteria use lactate to maintain gut health, while some pathogenic bacteria exacerbate disease through excess lactate production. Emerging therapeutic potential of lactate, including lactate dehydrogenase inhibitors, offers promising treatment avenues. This review provides a comprehensive overview of lactate’s complex role in health and disease, emphasizing the need for targeted strategies to harness its benefits while mitigating its harmful effects.

## 1 Introduction

Lactate, first discovered in 1780, was long considered a metabolic waste product generated under hypoxic conditions. However, the lactate shuttle hypothesis redefined its role, highlighting its function in oxidative metabolism, gluconeogenesis, and cellular signaling. Contrary to early misconceptions, lactate is actively produced and utilized under aerobic conditions, serving as a key modulator of systemic metabolism. It is transported via monocarboxylate transporters (MCTs) and signals through G protein-coupled receptor 81 (GPR81) (Lee, 2021).

**FIGURE 1:**
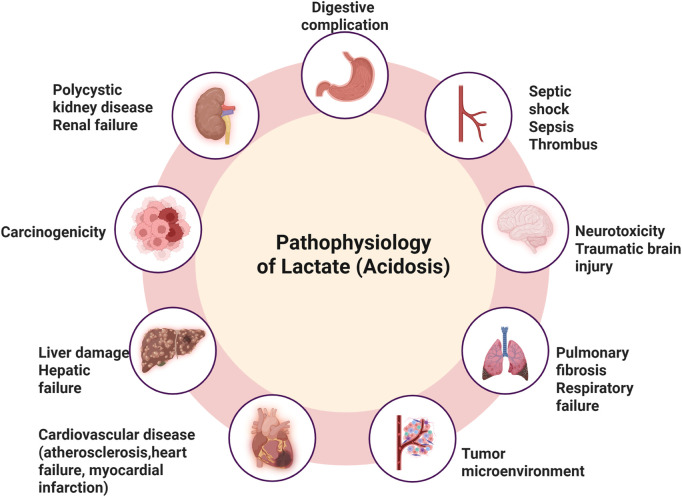
Pathophysiological impacts of lactate accumulation and acidosisElevated lactate levels leading to acidosis are implicated in various systemic complications, including digestive complications, septic shock, sepsis, and thrombus formation. Neurological effects include neurotoxicity and traumatic brain injury, while pulmonary consequences involve pulmonary fibrosis and respiratory failure. Lactate-driven acidosis also contributes to tumor microenvironment remodeling, promoting carcinogenicity. Furthermore, it is associated with cardiovascular diseases (such as atherosclerosis, heart failure, and myocardial infarction), liver damage leading to hepatic failure, and renal impairments, including polycystic kidney disease and renal failure.

In exercise physiology, lactate accumulation has traditionally been linked to muscle fatigue, though it is now recognized as an important energy source and metabolic regulator. In oncology, Otto Warburg’s 1920s discovery of aerobic glycolysis, where tumors ferment glucose into lactate even in oxygen-rich conditions, revealed lactate’s role in tumor metabolism. This Warburg effect is also observed in various non-cancerous conditions, including inflammatory diseases and metabolic disorders. Beyond cancer, lactate accumulation contributes to pathophysiological processes in pulmonary hypertension, pulmonary fibrosis, heart failure, atherosclerosis, and polycystic kidney disease (Figure 1). It is involved in stress responses during trauma, infection, and myocardial infarction. Moreover, lactate plays a crucial role in epigenetic regulation through lactylation, a posttranslational modification of histones that influences gene expression, particularly in inflammation and tumor progression (Mandadzhiev, 2025).

This review examines lactate’s diverse functions in health and disease, emphasizing its roles in metabolic reprogramming, immune modulation, and disease progression, underscoring the need for further research on its broader physiological and pathological implications.

## 2 Lactate production and metabolism

Lactate synthesis occurs in the cytoplasm via lactate dehydrogenase (LDH), which catalyzes the conversion of pyruvate to lactate while regenerating NAD^+^ from NADH. This reaction is crucial for sustaining glycolysis under anaerobic or hypoxic conditions, ensuring ATP production when oxidative phosphorylation is impaired. The direction of this reversible reaction depends on oxygen availability, intracellular NADH/NAD^+^ ratios, and metabolic demands (Lee, 2021).

Under normoxic conditions, pyruvate enters the mitochondria for oxidation via the tricarboxylic acid (TCA) cycle, producing ATP efficiently. However, during hypoxia, intense exercise, or pathological conditions like ischemia and cancer, oxidative metabolism is restricted, leading to NADH accumulation. To maintain glycolytic flux, LDH reduces pyruvate to lactate, restoring NAD^+^ for continued glycolysis (Liu et al., 2025).

Lactate synthesis is regulated by enzyme isoforms, substrate availability, and signaling pathways. LDH exists as isoenzymes composed of LDHA and LDHB subunits, with tissue-specific functions. LDHA, predominant in glycolytic tissues like skeletal muscle, favors lactate production, while LDHB, abundant in oxidative tissues like the heart, facilitates lactate oxidation. The NADH/NAD^+^ ratio is a key determinant of LDH activity, with high NADH levels promoting lactate formation (Wang et al., 2018).

Cellular signaling also modulates lactate metabolism. Hypoxia-inducible factor 1-alpha (HIF-1α) upregulates glycolytic enzymes and LDHA under low oxygen conditions, enhancing lactate production. Adrenergic stimulation during exercise accelerates glycolytic flux, increasing lactate accumulation, whereas insulin and other metabolic regulators influence lactate utilization. Lactate is not merely a result of metabolism but a key intermediary in energy production. It is transported via MCTs and serves as a metabolic substrate in various tissues, supporting gluconeogenesis in the liver and oxidative metabolism in the heart, highlighting its role in systemic metabolic flexibility (Zhang T. et al., 2024).

### 2.1 Physiological processes

Once regarded merely as a metabolic waste product generated during anaerobic glycolysis, lactate has emerged as a pivotal metabolic and signaling molecule integral to diverse physiological and pathological processes. Traditionally associated with muscle fatigue during intense exercise, lactate’s role extends far beyond a simple byproduct; it serves as an essential energy substrate, a regulator of cellular metabolism, and a mediator of intercellular communication (Vavřička et al., 2024).

In cancer biology, lactate’s significance is profound. Tumors commonly exhibit a metabolic shift known as aerobic glycolysis or the “Warburg effect,” where glucose is preferentially converted to lactate despite sufficient oxygen availability (Zhong et al., 2022). This metabolic reprogramming leads to lactate accumulation within the tumor microenvironment, which promotes cancer cell proliferation, angiogenesis, and metastasis (de la Cruz-López et al., 2019). Elevated lactate also contributes to immune evasion by creating an acidic microenvironment that suppresses cytotoxic T cell and natural killer cell activity, facilitating tumor progression (Gu et al., 2025). Research has demonstrated that lactate acts through signaling pathways such as HIF-1α stabilization and GPR81 activation, further reinforcing cancer cell survival and immune modulation (Chen et al., 2025). Beyond oncology, lactate is implicated in several chronic inflammatory and metabolic diseases (Lee, 2021). For instance, in pulmonary hypertension and pulmonary fibrosis, elevated lactate levels correlate with vascular remodeling and fibrotic processes (Peng et al., 2025). Studies indicate that lactate stimulates fibroblast activation and extracellular matrix deposition, contributing to disease progression (Caslin et al., 2021). In heart failure and atherosclerosis, abnormal lactate metabolism is associated with impaired mitochondrial function and chronic inflammation. Elevated circulating lactate in these conditions reflects underlying tissue hypoxia and metabolic stress, exacerbating cardiac dysfunction and vascular pathology (Zymliński et al., 2018; Zhu et al., 2024). Polycystic kidney disease (PKD) also features altered lactate metabolism. Experimental models show that lactate accumulation in cystic epithelial cells supports their proliferation and survival, contributing to cyst growth (Ghazi et al., 2019; Pagliarini and Podrini, 2021). Similarly, metabolic disorders such as type 2 diabetes exhibit dysregulated lactate production, linking it to insulin resistance and chronic low-grade inflammation (Nareika et al., 2005; Wu et al., 2016). Lactate involvement extends to acute stress responses, including trauma, infection, myocardial infarction, and ischemia-reperfusion injury (Wang et al., 2025). Here, lactate serves as both a metabolic fuel for damaged tissues and a signaling molecule triggering adaptive responses. Elevated lactate levels in sepsis, for example, are a prognostic marker reflecting tissue hypoperfusion and metabolic derangement (Liu et al., 2024).

A groundbreaking discovery expanding lactate role in disease is its function in epigenetic regulation through histone lactylation. This recently identified posttranslational modification involves the addition of lactate-derived groups to histone lysine residues, altering chromatin structure and gene expression. Histone lactylation has been shown to modulate inflammatory gene expression in macrophages, promoting resolution of inflammation, while also contributing to oncogenic gene programs in cancer cells. This epigenetic mechanism underscores lactate’s capacity to link metabolic states with long-term changes in cellular phenotype and disease outcomes (Zhang et al., 2019).

Collectively, lactate acts as a critical mediator of metabolic adaptation, immune regulation, and pathophysiological remodeling across a spectrum of diseases. Its dual roles as an energy substrate and signaling molecule highlight the intricate connections between metabolism and cellular function. Ongoing research aims to eKMCTlucidate therapeutic strategies targeting lactate metabolism and signaling, offering promising avenues for treating cancer, inflammatory disorders, cardiovascular diseases, and metabolic syndromes. Understanding lactate’s diverse physiological and pathological functions is essential for developing novel interventions to modulate disease progression and improve patient outcomes (Li et al., 2022).

### 2.2 Key sites of lactate production

During vigorous exercise, skeletal muscles rely on anaerobic glycolysis, resulting in substantial lactate accumulation. Once thought to be simply a product of glycolysis, lactate is now recognized as a vital signaling molecule and energy source. It can be shuttled to the liver for gluconeogenesis or oxidized in oxidative muscle fibers and the heart. Lactate also modulates gene expression related to mitochondrial biogenesis and angiogenesis, supporting muscle adaptation during endurance training (Mandadzhiev, 2025).

Erythrocytes, which lack mitochondria, depend entirely on anaerobic glycolysis for ATP production and continuously produce lactate. Recent studies highlight the role of MCTs in lactate transport, essential for maintaining acid-base balance. Erythrocyte-derived lactate significantly influences systemic metabolism, particularly in the heart and brain, and emerging evidence suggests its levels may serve as biomarkers for metabolic disorders such as diabetes and sepsis (Zhang T. et al., 2024).

In the brain, lactate plays a vital role through the astrocyte-neuron lactate shuttle. Astrocytes convert glucose into lactate, which neurons utilize during heightened activity. Lactate supports synaptic plasticity, learning, and memory by enhancing brain-derived neurotrophic factor (BDNF) signaling. Impaired lactate metabolism is linked to neurodegenerative diseases like Alzheimer’s and Parkinson’s, highlighting its therapeutic potential in cognitive disorders (Beard et al., 2022).

Cancer cells exhibit the Warburg effect, favoring glycolysis even in oxygen-rich environments, leading to lactate accumulation. This promotes immune evasion, angiogenesis, metastasis, and epigenetic changes that drive tumor aggressiveness. Lactate transporters such as MCT1 and MCT4 are emerging targets, with their inhibition shown to reduce tumor progression in breast cancer models. Modulating lactate metabolism offers a promising strategy for cancer therapy (Zhong et al., 2022).

### 2.3 Lactate metabolism

**FIGURE 2:**
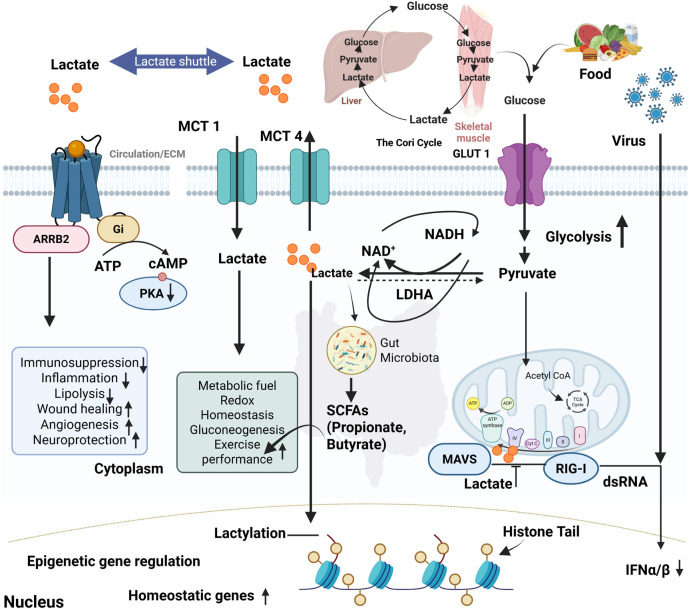
Lactate metabolism, signaling pathways, and its systemic impact on homeostasis and immunity. Food intake and viral infections drive glycolysis, leading to pyruvate and lactate production. Lactate is transported across membranes by MCT1& MCT4 and shuttled between tissues such as skeletal muscle and liver via the Cori cycle. Intracellularly, lactate modulates signaling through ARRB2-Gi protein-coupled pathways, reducing cAMP/PKA activity and influencing immunosuppression, inflammation, lipolysis, wound healing, angiogenesis, and neuroprotection. Lactate also serves as a metabolic fuel, supporting redox balance, gluconeogenesis, and exercise performance. In the cytoplasm, lactate-derived short-chain fatty acids (SCFAs) from gut microbiota contribute to systemic homeostasis. Lactate can inhibit mitochondrial antiviral signaling (MAVS) and RIG-I-mediated antiviral responses, reducing IFN-α/β production. Additionally, lactate enters the nucleus and promotes histone lactylation, driving epigenetic regulation of homeostatic gene expression.

Lactate is transported to the liver, heart, and oxidative muscle fibers, where it converts to pyruvate and enters the TCA cycle for ATP production (Wang et al., 2018). The lactate shuttle hypothesis, first introduced by Brooks (1985), describes lactate’s movement between glycolytic and oxidative tissues as a fuel source. The heart preferentially utilizes lactate as an energy substrate, with lactate oxidation playing a substantial role in myocardial energy production during both resting and stressed states (Brooks, 2021; Zhang H. et al., 2024) (Table 1). Through the Cori cycle, lactate is transported to the liver for gluconeogenesis (Figure 2), forming glucose that is released into the bloodstream (Melkonian et al., 2023). Lactate homeostasis is maintained through MCTs 1–4, enabling rapid cellular exchange (Figure 2). Elevated lactate levels (lactic acidosis) signal metabolic dysregulation. Lactate’s clearance mechanisms include oxidation via the TCA cycle, renal excretion through sodium/lactate transporters (Slc5a12 and Slc5a8), and microbiome incorporation (Lee, 2021). In a study, it has been demonstrated that hepatic gluconeogenesis is essential for glucose homeostasis during exercise and fasting (Meyer et al., 2002). The kidneys also contribute significantly, accounting for approximately 40% of systemic glucose production during prolonged fasting. Recent research challenges the traditional view of lactate as a glycolytic byproduct, showing that it directly fuels mitochondrial oxidative phosphorylation. Lactate enters mitochondria via the mitochondrial lactate oxidation complex (mLOC) and converts into pyruvate for ATP generation (Brooks et al., 2022). Hashimoto et al. (2008) provided compelling evidence that lactate oxidation occurs within mitochondria, highlighting its role in both normal and pathological states such as cancer and neurodegeneration (Hashimoto et al., 2008).

**TABLE 1:** Beneficial role of lactate in human health and underlying mechanisms.

Beneficial role	Mechanism	Relevant system/Organ	References
Energy substrate	Provides an efficient fuel source for muscles, heart, and brain	Muscle, Heart, Brain	Hashimoto et al. (2008)
Muscle adaptation	Enhances mitochondrial biogenesis and oxidative capacity	Skeletal muscle	Nalbandian et al. (2017)
Neuroprotection	Supports neuronal metabolism, prevents excitotoxicity, and enhances synaptic plasticity	Brain	Suzuki et al. (2011)
Immune modulation	Regulates macrophage polarization, suppresses inflammation, and supports Treg function	Immune system	Zhang et al. (2024b)
Angiogenesis and wound healing	Stimulates vascular endothelial growth factor (VEGF) production	Endothelial cells, Skin	Hunt et al. (2007)
Gluconeogenesis	Serves as a precursor for glucose production via the Cori cycle	Liver	Rogatzki et al. (2015)
Lipid metabolism regulation	Modulates free fatty acid (FFA) oxidation and storage	Adipose tissue	Bergman et al. (1999)
Exercise recovery	Reduces muscle fatigue, maintains pH balance, and promotes lactate shuttling	Muscle	Gladden (2004)
Cancer metabolism	Supports tumor cell survival and immune evasion in the tumor microenvironment	Tumor microenvironment	Fischer et al. (2007)
Cardioprotection	Preferred substrate over glucose in ischemic conditions, reducing oxidative stress	Heart	Chatham (2002)

### 2.4 Physiological roles of lactate

Lactate serves as an efficient energy source, particularly in oxidative tissues like the heart and brain. The heart preferentially oxidizes lactate over glucose, emphasizing its role in cardiac metabolism (Lee, 2021). Schurr et al. (1999) demonstrated that lactate is a primary energy source for neurons, reinforcing its significance beyond glycolysis (Schurr et al., 1999). Lactate regulates gene expression, angiogenesis, and immune responses (Figure 2). Lactate promotes the stabilization of HIF-1α by inhibiting prolyl hydroxylase activity. This stabilization enhances HIF-1-mediated transcription of glycolytic enzymes and VEGF, supporting metabolic adaptation and angiogenesis under hypoxic or pseudohypoxic conditions (Li et al., 2022). A study demonstrated that lactate accumulation under hypoxic conditions enhances VEGF expression, facilitating angiogenesis and tissue survival. Lactate accumulation during high-intensity exercise leads to metabolic acidosis but is rapidly cleared and utilized as an energy source. It aids recovery by replenishing glycogen stores and supporting mitochondrial respiration (Mandadzhiev, 2025). Gladden and Hogan (2006) showed that lactate metabolism during and after exercise is crucial for sustaining muscle function and preventing fatigue (Gladden and Hogan, 2006). Lactate is essential for brain metabolism. The astrocyte-neuron lactate shuttle theory suggests that astrocytes produce lactate, which neurons use as an energy source. Lactate enhances synaptic plasticity and memory formation (Hu et al., 2021). Pellerin et al. (1994) identified lactate as a key neuronal energy substrate, while another study showed that lactate enhances long-term memory formation in animal models (Pellerin et al., 1994). Lactate influences immune cell metabolism and function, regulating macrophage polarization. It promotes anti-inflammatory M2 macrophages while inhibiting pro-inflammatory M1 macrophages. This is particularly relevant in cancer metabolism and immune evasion (Zhang T. et al., 2024). In research study showed that lactate accumulation in tumors promotes M2 macrophage polarization, contributing to immune suppression and tumor progression (Zhong et al., 2022). Cancer cells convert glucose to lactate even in oxygen-rich conditions, a phenomenon known as the Warburg effect. Lactate accumulation in the tumor microenvironment promotes angiogenesis, immune evasion, and metastasis. Warburg first described this metabolic shift, and more recent studies highlighted how lactate metabolism supports tumor growth and resistance to therapy. Lactate is a vital metabolic intermediate with diverse roles beyond glycolysis. Its involvement in energy production, signaling, neuroprotection, and immune modulation underscores its physiological significance. The traditional perception of lactate as a waste product has evolved into its recognition as a key player in metabolic homeostasis (Nath and Balling, 2024).

In summary (Table 1), Lactate, an energy substrate, efficiently fuels muscles, the heart, and the brain, especially under high-demand conditions (Hashimoto et al., 2008). In skeletal muscle, lactate promotes adaptation by enhancing mitochondrial biogenesis and oxidative capacity, thereby improving endurance and performance (Nalbandian et al., 2017). In the brain, it supports neuronal metabolism, prevents excitotoxicity, and enhances synaptic plasticity, contributing to neuroprotection (Suzuki et al., 2011). Lactate also plays a crucial role in immune modulation by regulating macrophage polarization, suppressing inflammation, and supporting regulatory T cell function (Zhang T. et al., 2024). Furthermore, it stimulates angiogenesis and wound healing by promoting vascular endothelial growth factor (VEGF) production in endothelial cells and skin (Hunt et al., 2007). In the liver, lactate serves as a precursor for gluconeogenesis via the Cori cycle, helping to maintain blood glucose levels (Rogatzki et al., 2015). Its influence extends to lipid metabolism, where it modulates free fatty acid oxidation and storage in adipose tissue (Bergman et al., 1999). During exercise recovery, lactate helps reduce muscle fatigue, maintain pH balance, and facilitate lactate shuttling between tissues (Gladden, 2004). Interestingly, in the tumor microenvironment, lactate supports cancer cell survival and immune evasion (Fischer et al., 2007). Lastly, in the heart, lactate serves as a preferred substrate over glucose during ischemic conditions, offering cardioprotection by reducing oxidative stress (Chatham, 2002). Collectively, these findings highlight lactate’s multifaceted and beneficial roles across various organs and systems, challenging its outdated reputation as merely a metabolic waste product. In the following section, we provide a detailed explanation of key aspects highlighted in (Table 1).

## 3 The boon: beneficial role of lactate

### 3.1 Lactate as an energy source

Glycolysis, a key metabolic pathway, processes glucose to generate ATP and essential precursors for cellular functions. While glycolysis contributes only ∼6% of cellular ATP via substrate-level phosphorylation, its regulation by enzymes such as hexokinase (HEX), phosphofructokinase (PFK), and pyruvate kinase (PK) is crucial. Pyruvate dehydrogenase (PDH) and pyruvate carboxylase (PC) further link glycolysis to mitochondrial metabolism. Traditionally considered a waste product of anaerobic metabolism, lactate is now recognized as a critical energy source. It is converted to pyruvate by lactate dehydrogenase (LDH) and utilized by muscles, the heart, and the brain for oxidative phosphorylation, particularly during exercise or metabolic stress (Chaudhry and Varacallo, 2023).

Lactate, first identified in sour milk through microbial fermentation, becomes the predominant metabolite in mammals when oxygen and ATP demands surpass supply. Contrary to its reputation as a metabolic waste product, lactate is now understood as a key circulating carbohydrate fuel. By balancing the NADH/NAD+ ratio, lactate serves as both an energy substrate and a redox buffer, facilitating metabolic flexibility. This shift in perspective redefines lactate’s role in energy metabolism (Li et al., 2022).

### 3.2 Lactate as a preferred fuel

During physical exertion, skeletal muscles and the heart preferentially utilize lactate over glucose and fatty acids. In the brain, the Astrocyte-Neuron Lactate Shuttle (ANLS) facilitates lactate transfer from astrocytes to neurons, promoting energy metabolism and neuroprotection (Cauli et al., 2023). Recent findings challenge the notion that lactate is merely converted to pyruvate in the cytosol; instead, it is processed in mitochondria via the Mitochondrial Lactate Oxidation Complex (mLOC), particularly in high-energy-demand tissues (Chen et al., 2016).

In resting humans, the lactate-to-pyruvate (L/P) ratio is ∼10, increasing to ∼500 during moderate exercise, underscoring lactate’s role in metabolic adaptation. Major lactate utilization pathways include intramuscular oxidation, cardiac uptake, and hepatic gluconeogenesis (Liu et al., 2025).

### 3.3 Lactate and lipid metabolism

An inverse relationship exists between lactate and plasma free fatty acids (FFAs) during exercise. Lactate inhibits lipolysis in adipose tissue via Hydroxycarboxylic Acid Receptor 1 (HCAR1), modulating cAMP and CREB signaling pathways. Additionally, lactate impacts mitochondrial fatty acid oxidation by increasing acetyl-CoA and malonyl-CoA levels, inhibiting β-oxidation (Liu et al., 2009).

Lactate-induced secretion of Transforming Growth Factor Beta 2 (TGF-β2) from adipose tissue enhances glucose tolerance, highlighting its role in interorgan metabolic communication. These findings emphasize lactate’s dual role in acute metabolic regulation and long-term adaptation (Liu et al., 2025).

### 3.4 Lactate in brain energy metabolism and neuroprotection

Neurons rely on astrocytes for metabolic support. While glucose remains the primary fuel, lactate serves as an essential alternative, particularly during hypoglycemia or ischemic stress. Lactate supplementation enhances recovery from traumatic brain injury (TBI) and supports memory formation by modulating epigenetic mechanisms and brain-derived neurotrophic factor (BDNF) expression. Lactate metabolism is altered in neurodegenerative diseases like Alzheimer’s and Parkinson’s, with reduced lactate transport linked to cognitive decline. Enhancing lactate availability may offer therapeutic benefits in restoring metabolic balance and neuroprotection (Beard et al., 2022).

### 3.5 Lactate as a cellular signal and epigenetic modulator

Beyond metabolism, lactate functions as a signaling molecule influencing angiogenesis, tissue repair, and gene expression. It activates G-protein coupled receptors such as HCAR1, regulating neuroprotection and lipid metabolism. A novel post-translational modification, histone lactylation, links lactate to gene regulation (Figure 2). Increased lactate production under hypoxia or bacterial infections drives histone lactylation, influencing macrophage polarization, tumor progression, and inflammation resolution. These positions lactate as a crucial metabolic and epigenetic regulator with broad physiological and pathological implications (Zhang et al., 2019).

### 3.6 Lactate in hypoxia and cancer metabolism

Lactate stabilizes HIF-1α, promoting angiogenesis and metabolic adaptation under low-oxygen conditions. Lactate signaling plays a central role in tumor metabolism and immune evasion; thus, its targeting represents an emerging avenue for cancer therapeutics (Gu et al., 2025).

### 3.7 Lactate in exercise adaptation and immune modulation

Lactate modulates mitochondrial biogenesis and endurance adaptations through AMP-activated protein kinase (AMPK) and PGC-1α signaling. It also influences immune responses, shaping macrophage polarization and cytokine production, with implications for inflammation, metabolism, and disease progression (Takeda et al., 2022).

## 4 Gut microbiota: fuel or metabolize excess lactate

### 4.1 Lactate production by gut bacteria

Lactic Acid Bacteria (LAB) have adapted to survive in different environments, including the human gut, by relying primarily on fermentation for energy production. Unlike many other bacteria, LAB cannot perform respiration because they lack functional cytochromes, which are essential for oxidative metabolism. Instead, they obtain energy by breaking down sugars into lactic acid, which acidifies their surroundings and helps them outcompete harmful bacteria (Wang et al., 2020).

LAB have evolved unique metabolic pathways that enable their survival in diverse environments, particularly within the human gut. Among these pathways, amino acid deamination serves as a crucial mechanism for energy production, allowing LAB to thrive in nutrient-poor conditions by converting amino acids into usable energy. Additionally, acid decarboxylation plays a fundamental role in maintaining pH balance, which is essential for LAB survival in acidic environments such as the gastrointestinal tract (Feng and Wang, 2020).

Beyond their metabolic adaptability, LAB engage in intricate interactions with host cells, significantly influencing gut health and immune function. Through cross-talk with the host, LAB can modulate intestinal gene expression, thereby impacting digestive processes and immune responses. Furthermore, LAB contributes to gut homeostasis by producing bioactive compounds, such as gamma-aminobutyric acid (GABA), which not only relaxes gut smooth muscles but also has broader implications for mood regulation. Another essential feature of LAB is their antimicrobial effect, primarily mediated by lactic acid production. It acidifies the gut environment and inhibits the growth of pathogenic bacteria, thereby fostering a balanced microbiome (Yu et al., 2024).

Lactate plays a dual role in gut health. On the one hand, it serves as an important metabolic intermediate, supporting the growth of lactate-consuming bacteria, which convert it into beneficial SCFAs (Table 4). On the other hand, excessive lactate accumulation can lead to acidosis, which disrupts microbial stability. This phenomenon is well-documented in ruminants, where diets rich in fermentable carbohydrates can promote the overgrowth of lactate-producing bacteria, causing a dangerous drop in pH and leading to metabolic disorders such as lactic acidosis. In the human gut, lactate metabolism is tightly regulated through cross-feeding interactions between different bacterial species (Yu et al., 2024).

### 4.2 Lactate utilization by gut microbes

#### 4.2.1 Butyrate production

**FIGURE 3:**
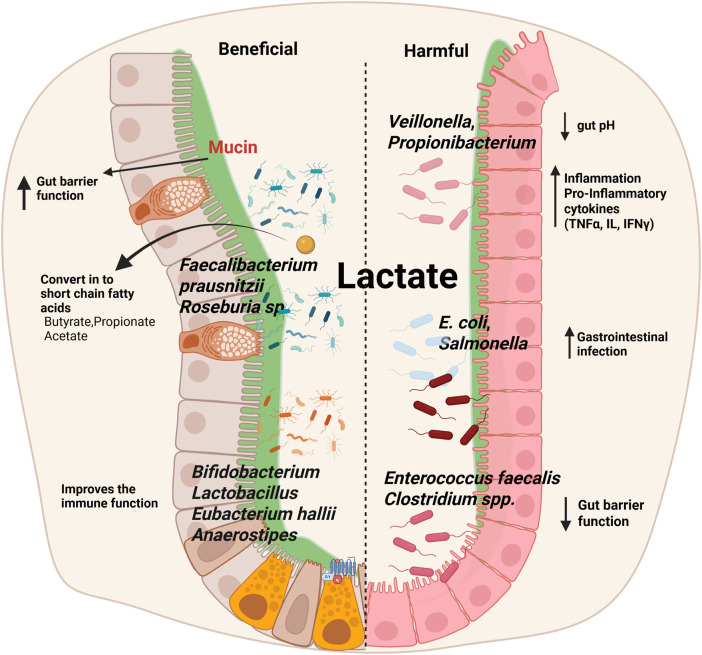
Impact of lactate on gut microbiota: balancing beneficial and harmful effects. Lactate influences gut microbiota composition, supporting beneficial bacteria such as *Faecalibacterium prausnitzii*, *Roseburia* spp., *Bifidobacterium*, *Lactobacillus*, *Eubacterium hallii*, and *Anaerostipes*, which enhance gut barrier integrity, improve immune function, and produce SCFAs like butyrate, propionate, and acetate. In contrast, elevated lactate levels can promote the growth of harmful bacteria, including *Veillonella*, *Propionibacterium*, *E. coli*, *Salmonella*, *Enterococcus faecalis*, and *Clostridium* spp., leading to reduced gut pH, increased inflammation via pro-inflammatory cytokines (TNF-α, ILs, IFN-γ), impaired gut barrier function, and a higher risk of gastrointestinal infections.

Certain members of the phylum Firmicutes, particularly species belonging to the genera *Anaerobutyricum* and *Anaerostipes*, are capable of converting lactate into butyrate, a SCFA with well-established health benefits. This conversion typically occurs via a cross-feeding mechanism that requires acetate as a co-substrate. Butyrate is a critical energy source for colonocytes, where it enhances intestinal barrier function, regulates inflammatory pathways, and modulates the gut immune response. Additionally, butyrate has been associated with anti-inflammatory effects through its role in the inhibition of histone deacetylases (HDACs), contributing to immune homeostasis within the gut mucosa (Figure 3) (Louis et al., 2022).

#### 4.2.2 Propionate formation

Lactate can also be metabolized into propionate via multiple biochemical pathways, with distinct microbial taxa utilizing different metabolic route.

#### 4.2.3 Acrylate pathway

This pathway involves the direct conversion of lactate into propionate and is employed by bacterial species such as *Coprococcus catus* (Reichardt et al., 2014) and *Megasphaera elsdenii* (Hino and Kuroda, 1993).

#### 4.2.4 Succinate pathway

In this pathway, lactate is first converted to succinate, which is subsequently metabolized into propionate. This process is utilized by species within the genus *Veillonella* (Reichardt et al., 2014).

#### 4.2.5 1,2-Propanediol pathway

An alternative route involves the conversion of lactate into 1,2-propanediol, which can then be further metabolized into propionate by species such as *Limosilactobacillus reuteri* and *Anaerobutyricum hallii* (Niu et al., 2019).

Propionate serves multiple physiological functions in the host, including modulating gluconeogenesis in the liver, influencing appetite regulation, and exerting anti-inflammatory properties through interactions with gut epithelial and immune cells (Louis et al., 2022).

#### 4.2.6 Acetate production and sulfate reduction

Certain *Proteobacteria*, including species within the genus *Desulfovibrio*, are capable of metabolizing lactate into acetate through dissimilatory sulfate reduction. This process is coupled with the production of hydrogen sulfide (H_2_S), a metabolite that, at excessive levels, has been implicated in gut epithelial damage and inflammatory conditions such as inflammatory bowel disease (IBD). While low concentrations of H_2_S may have physiological roles in cell signaling, its accumulation can lead to mucosal toxicity and alterations in gut microbiota composition (Rey et al., 2013).

The microbial conversion of lactate into butyrate, propionate, and acetate plays a pivotal role in gut ecosystem stability and host physiology. Cross-feeding interactions between lactate-producing and lactate-utilizing bacteria regulate metabolite flux, influencing gut barrier integrity, immune function, and metabolic homeostasis (Dordević et al., 2020).

#### 4.2.7 Cross-feeding among microbes

Bacterial cross-feeding plays a crucial role in shaping SCFA production and optimizing substrate utilization in the gut. These interactions enhance microbial diversity, prevent metabolic imbalances, and contribute to gut health by maintaining a stable environment. *Bifidobacterium* produces acetate when fermenting dietary fibers such as oligofructose, which is then utilized by butyrate-producing bacteria like *Faecalibacterium prausnitzii* and *Roseburia* sp. to produce butyrate. Lactate-producing bacteria like *Bifidobacterium* and *Lactobacillus* produce lactate during carbohydrate fermentation, which can be converted into butyrate by *Eubacterium hallii* and *Anaerostipes* to prevent acid build-up and contribute to gut health. Propionate-producing bacteria (*Veillonella*, *Propionibacterium*) convert lactate into propionate, playing a role in glucose metabolism and satiety (Figure 3) (Culp and Goodman, 2023).

### 4.3 Implications for intestinal homeostasis

Lactate plays a significant role in gut health, immunity, and disease prevention. If lactate-consuming bacteria are insufficient, lactate can accumulate, leading to a drop in pH and potential gut dysbiosis. Excess lactate has been linked to inflammatory bowel diseases (IBD), irritable bowel syndrome (IBS), and metabolic disorders (Wang et al., 2020). Colonocytes (intestinal cells) utilize lactate to produce butyrate through cross-feeding by bacteria such as *Eubacterium* and *Anaerostipes*, which strengthen the gut lining and support overall intestinal health. Lactate lowers the pH, inhibiting harmful bacteria (*Escherichia coli*, *Salmonella*) while promoting beneficial microbes (*Bifidobacteria*, *Akkermansia*) (Zhao et al., 2024). Additionally, lactate helps regulate immune responses by modulating immune cells and reducing inflammatory cytokines (TNF-α, IL-6), preventing excessive inflammation (Llibre et al., 2025).

Recent studies highlight the pivotal role of lactate and its receptor, GPR81, in regulating intestinal homeostasis and immune responses, particularly in inflammatory bowel diseases (IBD) (Ranganathan et al., 2018). Understanding these metabolic pathways and their microbial contributors provides valuable insight into potential probiotic interventions aimed at promoting gut health and preventing dysbiosis-associated diseases (Li et al., 2022).

## 5 Lactate dehydrogenase (LDH) inhibitors: plant-based and synthetic

Lactate dehydrogenase (LDH) plays a crucial role in anaerobic glycolysis by catalyzing the reversible conversion of pyruvate to lactate, coupled with the regeneration of NAD^+^. In cancer, this pathway is hijacked even under aerobic conditions, a phenomenon known as the Warburg effect, whereby cancer cells preferentially convert glucose to lactate despite sufficient oxygen. This metabolic reprogramming supports rapid cell proliferation by sustaining glycolytic flux, maintaining redox balance, and promoting survival in the tumor microenvironment. LDH, particularly the LDH-A isoform, is thus considered a key metabolic enzyme and a promising therapeutic target in cancers (Valvona et al., 2016; Kim et al., 2019) and infectious diseases like malaria (Possemiers et al., 2021). LDH belongs to the 2-hydroxyacid oxidoreductase family and is widely found in animals, microorganisms, yeasts, and plants. The Table 2 summarizes various plant-based LDH inhibitors, highlighting their mechanisms of action and implications for human health (Table 2). Natural compounds such as pentagalloyl glucose from Rhus chinensis, polyphenols (including quercetin, EGCG, curcumin, and resveratrol), and berberine target LDH through different mechanisms, such as non-competitive inhibition, binding to the enzyme’s active or coenzyme sites, and suppression of LDH expression. These inhibitors show potential in cancer therapy by disrupting tumor metabolism, overcoming drug resistance, and modulating lactate production. Additionally, some compounds like those from Polygala tenuifolia demonstrate neuroprotective effects in ischemic stroke, while others, such as rutin and amentoflavone from Selaginella doederleinii, offer potential treatments for metabolic disorders. Overall, plant-based LDH inhibitors present promising therapeutic avenues for cancer, metabolic diseases, and neuroprotection (Table 2).

**TABLE 2:** Summarizes the plant-based and synthetic LDH inhibitors.

Category	LDH inhibitor	Role/Mechanism	Implications for human health	References
Plant-Based Inhibitors	*Rhus chinensis*	Pentagalloyl glucose (PGG) inhibits LDH non-competitively, reducing lactate in cancer cells	Potential cancer therapy by disrupting metabolic processes in tumors	Mendonca et al. (2021)
	*Polygala tenuifolia*	Identified five LDH inhibitors, a potential treatment for ischemic stroke	Neuroprotective effects in ischemic stroke	Shen et al. (2025)
	Rutin	Binds to the coenzyme site of LDH, inhibiting its activity in a dose-dependent manner	Modulates lactate production, useful in various metabolic disorders	Ding et al. (2024)
	*Selaginella doederleinii*	Amentoflavone and robustaflavone inhibit LDH	Potential treatment for metabolic disorders and cancer	Zhang et al. (2022)
	Polyphenols (Quercetin, EGCG, Curcumin, Resveratrol)	Binds to the LDH active site, reduces its activity, and suppresses LDH expression	Anti-cancer and anti-inflammatory effects, metabolic modulation	Han et al. (2023)
	Catechin	Inhibits lactate production and LDHA activity, overcomes 5FU resistance in cancer	Potential adjuvant therapy for drug resistance in cancer	Han et al. (2023)
	Berberine	Targets LDH-A, suppressing pancreatic cancer progression via AMPK/mTOR pathway	Potential cancer therapy, metabolic disorder treatment	Davoodvandi et al. (2024)
	Betulinic Acid	Binds strongly to LDH, reducing its activity, and exerting antitumor effects	Anti-cancer properties, modulates lactate production in tumors	Davoodvandi et al. (2024)
Synthetic Inhibitors	Pyrazole-based inhibitors	Block LDH enzymatic activity, developed through high-throughput screening	Potential cancer and metabolic disorder therapy	Rai et al. (2020)
	Galloflavin	Inhibits LDH-A and LDH-B, induces apoptosis in tumor cells	Selective anticancer agent, blocks aerobic glycolysis in tumors	Yao et al. (2022)
	Oxamate	Inhibits LDH-A in NSCLC cells, reduces ATP levels and induces apoptosis	Targeting LDH-A for cancer therapy, particularly in lung cancer	Li et al. (2020)
	GSK2837808A	Specific LDH-A inhibitor, reduces lactate secretion in TMJOA synovial fibroblasts	Potential treatment for joint disorders and metabolic dysfunctions	Alobaidi et al. (2023)
	FX-11 and AR-C155858	Combination therapy targeting LDHA and MCT1, reducing tumor cell proliferation	Effective in breast and colorectal cancer treatment	Alobaidi et al. (2023)
	N-hydroxyindole-based inhibitor (NHI-Glc-2)	Inhibits glycolysis and cell proliferation in cancer	Potential anticancer agent with antiglycolytic effects	Hassouni et al. (2020)
	Itraconazole, Atorvastatin, Posaconazole	Potential inhibitors of Plasmodium LDH, with posaconazole most effective	Treatment for malaria, particularly against Plasmodium LDH	Comandatore et al. (2022)
	Stiripentol	Antiepileptic drug, inhibits LDH, reducing seizures and epileptiform activity	Potential treatment for epilepsy, reducing lactate-related neuronal activity	Wheless and Weatherspoon (2025)

Beyond its metabolic role, LDH is an important diagnostic marker, as its sudden increase in serum levels often indicates acute disease conditions. High LDH levels are commonly observed in malignancies, megaloblastic anemia, myocardial infarction, liver disorders, hematological diseases, and skeletal muscle conditions. Due to its strong clinical significance, LDH measurement is widely used for disease diagnosis and monitoring (Gupta, 2022).

### 5.1 LDH in malaria and cancer

In *Plasmodium falciparum* (the parasite responsible for malaria), the enzyme pfLDH is essential for energy production, as the parasite relies on anaerobic glycolysis due to the absence of a citric acid cycle. Inhibitors of pfLDH could potentially lead to the death of the malaria parasite (Penna-Coutinho et al., 2011). Similarly, in cancer, human LDH isoform-5 (hLDH-5 or LDH-A) is upregulated in tumor cells, supporting the Warburg effect, where tumors rely more on anaerobic glycolysis than oxidative phosphorylation for energy. Targeting hLDH-5 could disrupt tumor growth and invasiveness (Alam et al., 2014).

## 6 The curse: potential detrimental effects of lactate

### 6.1 Lactic acidosis and metabolic dysregulation

While lactate serves as a critical metabolic intermediate, excessive accumulation can lead to lactic acidosis, a pathological condition characterized by a decrease in blood pH. This occurs in cases of severe hypoxia, sepsis, or mitochondrial dysfunction, where lactate clearance is impaired. Studies have shown that elevated lactate levels in critically ill patients are associated with poor outcomes due to systemic metabolic disturbances (Table 3) (Li et al., 2022). Recently, research highlighted the role of excessive lactate in disrupting cellular homeostasis, leading to impaired enzyme function and metabolic stress (Cai et al., 2024).

**TABLE 3:** Summary of the impact of excess lactate on human health and the mechanisms involved in disease progression.

Effect	Mechanism	Relevant organs	References
Cancer progression	Lactate supports tumor growth by driving enhanced glycolysis and suppressing immune responses	Tumors (Various organs)	de la Cruz-López et al. (2019)
Increased inflammation	Lactate accumulation can activate inflammatory pathways and cytokine release	Immune system, tissues	Llibre et al. (2025)
Metabolic disorders	High lactate levels contribute to metabolic dysfunction, affecting glucose and lipid metabolism	Liver, muscles, adipose tissue	Ishitobi et al. (2019)
Muscle fatigue	Accumulation of lactate in muscles leads to acidosis and impairs muscle contraction	Skeletal muscles	Chen et al. (2025)
Brain dysfunction	Lactate buildup in the brain may contribute to neurological diseases like epilepsy and Alzheimer’s	Brain (CNS)	Cai et al. (2022)
Cardiac stress	Elevated lactate in the heart can cause reduced oxygen availability, increasing the risk of ischemia	Heart	Fang et al. (2024)

Excessive lactate accumulation, as detailed in Table 3, has significant implications for human health by contributing to the progression of various diseases. In cancer, elevated lactate levels promote tumor growth through enhanced glycolysis and suppression of immune responses, affecting multiple organs (de la Cruz-López et al., 2019) (Table 3). Lactate buildup can also activate inflammatory pathways and cytokine release, leading to increased inflammation in the immune system and other tissues (Llibre et al., 2025) (Table 3). Metabolic disorders arise when high lactate concentrations disrupt glucose and lipid metabolism, particularly impacting the liver, muscles, and adipose tissue (Ishitobi et al., 2019). In skeletal muscles, lactate accumulation results in acidosis and impairs muscle contraction, contributing to muscle fatigue (Chen et al., 2025) (Table 3). The brain is also vulnerable, as lactate buildup may play a role in neurological diseases such as epilepsy and Alzheimer’s (Cai et al., 2022) (Table 3). Lastly, in the heart, elevated lactate can reduce oxygen availability and increase the risk of ischemia (Fang et al., 2024) (Table 3). This summary underscores the multifaceted and detrimental effects of excess lactate on various organ systems.

### 6.2 Tumor microenvironment and cancer progression

The Warburg effect, a hallmark of cancer metabolism, leads to high lactate production, which significantly alters the tumor microenvironment. Accumulated lactate promotes immune evasion, angiogenesis, and metastasis by modulating tumor-associated macrophages and regulatory T cells. Studies have shown that lactate enhances HIF-1α stabilization, upregulating VEGF expression and supporting tumor growth. In study showed that targeting lactate transporters, such as MCT1 and MCT4, can disrupt tumor metabolism and enhance the efficacy of cancer therapies (Zhong et al., 2022).

### 6.3 Cardiovascular disease and impaired cardiac function

Elevated lactate levels are frequently observed in cardiovascular diseases, especially in conditions like heart failure and ischemic injury, where tissue hypoxia impairs oxidative metabolism. The heart preferentially oxidizes lactate as an energy source under normal conditions; however, in pathological states, excessive lactate accumulation can contribute to acidosis and impaired myocardial function (Ouyang et al., 2023). A study illustrated that heart failure patients exhibit altered lactate metabolism, leading to reduced cardiac efficiency (Revelly et al., 2005). Moreover, lactate-driven inflammation in vascular endothelial cells has been implicated in atherosclerosis progression, highlighting the need for therapeutic strategies targeting lactate balance in cardiovascular health (Dong et al., 2021).

### 6.4 Neurological disorders and cognitive dysfunction

Dysregulated lactate metabolism has been implicated in neurodegenerative diseases, including Alzheimer’s, Parkinson’s, and multiple sclerosis. While lactate plays a neuroprotective role under physiological conditions, impaired lactate transport and utilization can contribute to neuronal energy deficits and oxidative stress (Yang et al., 2024). A research study demonstrated that disruptions in the astrocyte-neuron lactate shuttle lead to cognitive impairment (Suzuki et al., 2011). Furthermore, excessive lactate accumulation in the brain has been associated with neuroinflammation and excitotoxicity, exacerbating disease progression in conditions such as epilepsy and traumatic brain injury (Roh et al., 2023).

### 6.5 Muscular dystrophy and exercise intolerance

In individuals with muscular dystrophy and metabolic myopathies, lactate metabolism is often impaired, leading to exercise intolerance and muscle fatigue. Mutations affecting glycolytic enzymes or lactate transporters result in an inability to efficiently clear lactate, leading to muscle damage and weakness (Jeppesen et al., 2013). A study highlighted the role of lactate accumulation in muscle dysfunction, emphasizing its contribution to oxidative stress and mitochondrial impairment. Therapeutic approaches aimed at optimizing lactate metabolism are being explored to improve muscle function in dystrophic conditions (Tarnopolsky, 2018; Wen et al., 2025).

### 6.6 Lactate-producing bacteria harmful to human health

While many lactate-producing bacteria play beneficial roles in maintaining gut homeostasis, certain species can contribute to dysbiosis and inflammation when their growth is dysregulated (Louis et al., 2022). For instance, an overgrowth of *Lactobacillus* or *Enterococcus* species can lead to excessive lactate accumulation, which may exacerbate gastrointestinal conditions such as IBS, IBD, and even metabolic diseases like obesity. In these cases, the excess lactate can lower gut pH, leading to an imbalance in the microbiota and promoting the growth of pathogenic bacteria (Figure 3; Table 4) (Portincasa et al., 2024).

**TABLE 4:** Summarized table of microbiota showing their beneficial and harmful impacts on human health in the presence of lactate.

Bacteria	Category	Mechanism of lactate production	Impact on human health	Potential health effects	References
*Lactobacillus* spp. *(Lactobacillus acidophilus; Lactobacillus casei)*	Beneficial	Ferments carbohydrates to produce lactate	Helps maintain gut acidity, beneficial for digestion, and supports gut health by preventing pathogenic overgrowth	Prevents pathogen colonization, supports immune function, and promotes gut barrier integrity	Dempsey and Corr (2022)
*Bifidobacterium* spp.	Beneficial	Produces lactate from dietary fibers and oligosaccharides	Modulates gut microbiota, improves gut health, and has anti-inflammatory effects	Supports healthy digestion, reduces inflammation, and promotes the production of beneficial short-chain fatty acids	Victoria Obayomi et al. (2024)
*Faecalibacterium prausnitzii*	Beneficial	Converts carbohydrates into lactate and butyrate	Supports gut health and maintains a balanced microbiome	Enhances anti-inflammatory responses and strengthens gut barrier function	Martín et al. (2023)
*Lactobacillus plantarum*	Beneficial	Produces lactic acid by fermenting sugars in anaerobic conditions	Promotes gut barrier integrity and reduces inflammation	May help in managing irritable bowel syndrome (IBS) and other inflammatory conditions	Liu et al. (2021)
*Lactobacillus rhamnosus*	Beneficial	Ferments glucose to lactic acid, regenerating NAD+ for glycolysis	Strengthens gut microbiota and supports immune function	Reduces risk of respiratory infections and improves skin health	Rastogi and Singh (2022)
*Lactobacillus reuteri*	Beneficial	Convert sugars to lactic acid via the lactate dehydrogenase enzyme	Produces antimicrobial substances that inhibit pathogens	May reduce colic in infants and improve oral health	Singh et al. (2022)
*Streptococcus thermophilus*	Beneficial	Converts lactose to lactic acid during fermentation	Aids in lactose digestion and supports gut health	Reduces symptoms of lactose intolerance and improves gut microbiota	Saleem et al. (2024)
*Enterococcus faecium*	Beneficial	Produces lactic acid as a byproduct of carbohydrate metabolism	Competes with harmful bacteria in the gut	May reduce the risk of gastrointestinal infections	Vieco-Saiz et al. (2019)
*Pediococcus acidilactici*	Beneficial	Ferments sugars to lactic acid, lowering pH and inhibiting spoilage organisms	Used in food preservation and supports gut health	Enhances food safety and promotes gut microbiota balance	Todorov et al. (2022)
*Leuconostoc mesenteroides*	Beneficial	Produces lactic acid and other metabolites during carbohydrate fermentation	Contributes to food fermentation and supports gut health	Improves digestion and enhances the flavor of fermented foods	Kim et al. (2025)
*Weissella confusa*	Beneficial	Ferments sugars to lactic acid, producing antimicrobial compounds	Inhibits harmful bacteria and supports gut health	May reduce the risk of infections and improve gut microbiota	Petrariu et al. (2023)
*Oenococcus oeni*	Beneficial	Converts malic acid to lactic acid during malolactic fermentation	Used in winemaking and supports gut health	Enhances the flavor of wine and promotes gut microbiota balance	James et al. (2023)
*Carnobacterium maltaromaticum*	Beneficial	Produces lactic acid and bacteriocins during fermentation	Inhibits spoilage organisms in food and supports gut health	Enhances food safety and promotes gut microbiota balance	Bisht et al. (2024)
*Propionibacterium freudenreichii*	Beneficial	Produces lactic acid and propionic acid during carbohydrate fermentation	Supports gut health and produces vitamin B12	Enhances nutrient absorption and reduces gut inflammation	Tripathi et al. (2024)
*Streptococcus mutans*	Harmful	Produces lactate from sugars, especially sucrose	Key contributor to dental cavities and oral infections	Causes tooth decay and promotes plaque formation	Luo et al. (2024)
*Clostridium* spp.	Harmful	Ferments carbohydrates to produce lactate, often in excess	Overproduction of lactate in gut can lower pH, cause metabolic disturbances, and exacerbate gut inflammation	Contributes to conditions like colorectal cancer, IBS, and IBD.	Hou et al. (2022)
*Enterococcus faecalis*	Harmful	Produces lactate from sugars under anaerobic conditions	Associated with gut dysbiosis, promotes inflammation and infection, and is pathogenic in immunocompromised individuals.	Can lead to sepsis, urinary tract infections, and gut-related issues like IBS and IBD.	Chancharoenthana et al. (2023)
*Staphylococcus aureus*	Harmful	Anaerobic fermentation of glucose	Produces lactate, which can contribute to tissue damage and inflammation	Causes skin infections, pneumonia, and toxic shock syndrome	Taylor and Unakal (2023)
*Escherichia coli (E. coli)*	Harmful	Glycolysis followed by fermentation under oxygen-limited conditions	Lactate production can exacerbate inflammation in the gut	Causes food poisoning, urinary tract infections, and septicemia	Milani et al. (2017)
*Streptococcus pyogenes*	Harmful	Lactic acid fermentation via the Embden-Meyerhof pathway	Lactate can fuel bacterial growth and worsen tissue damage	Causes strep throat, scarlet fever, and necrotizing fasciitis	Stevens and Bryant (2022)
*Clostridium perfringens*	Harmful	Fermentation of pyruvate in anaerobic conditions	Lactate production contributes to the acidic environment, aiding bacterial survival	Causes gas gangrene and food poisoning	Juneja et al. (2023)
*Lactobacillus acidophilus*	Harmful	Homolactic fermentation, where glucose is converted entirely into lactate	Overproduction of lactate can lead to acidosis in certain conditions	Normally beneficial but can cause infections in immunocompromised individuals	Gao et al. (2022)
*Propionibacterium acnes*	Harmful	Fermentation of carbohydrates under anaerobic or oxygen-restricted conditions	Lactate production can contribute to inflammation in sebaceous glands	Associated with acne and other skin conditions	Gannesen et al. (2019)
*Helicobacter pylori*	Harmful	Adaptive metabolic pathways for survival in acidic stomach conditions	Lactate production can alter the stomach’s microenvironment, aiding bacterial colonization	Causes stomach ulcers and is linked to gastric cancer	Somiah et al. (2022)
*Klebsiella pneumoniae*	Harmful	Facultative anaerobic fermentation of sugars	Lactate production can worsen lung inflammation and tissue damage	Causes pneumonia, urinary tract infections, and septicemia	Liang et al. (2022)
*Pseudomonas aeruginosa*	Harmful	Lactate synthesis during anaerobic respiration	Lactate production supports biofilm formation, making infections harder to treat	Causes infections in wounds, lungs, and urinary tract	Moser et al. (2021)
*Enterococcus faecalis*	Harmful	Fermentation via the glycolytic pathway	Lactate production can contribute to biofilm formation and antibiotic resistance	Causes urinary tract infections, endocarditis, and bacteremia	Said et al. (2024)
*Bacteroides fragilis*	Harmful	Anaerobic glycolysis	Lactate production can promote bacterial survival in anaerobic environments	Causes abdominal infections and abscesses	Tufail and Schmitz (2024)
*Clostridium difficile*	Harmful	Lactic acid fermentation in low-oxygen environments	Lactate production can disrupt gut microbiota balance	Causes severe diarrhea and colitis	Sehgal and Khanna (2021)
*Mycobacterium tuberculosis*	Harmful	Modulation of metabolic pathways under hypoxic conditions	Lactate production can contribute to the acidic environment in infected tissues	Causes tuberculosis	Kiran and Basaraba (2021)
*Neisseria gonorrhoeae*	Harmful	Oxygen-limited glycolysis followed by fermentation	Lactate production can enhance bacterial survival in mucosal tissues	Causes gonorrhea	Llibre et al. (2021)
*Salmonella enterica*	Harmful	Facultative fermentation of carbohydrates	Lactate production can exacerbate gut inflammation and bacterial invasion	Causes foodborne illnesses and typhoid fever	Llibre et al. (2021)

The Table 4 highlights the beneficial roles of various gut bacteria in human health through their mechanisms of lactate production. *Lactobacillus* spp. (including L. acidophilus and L. casei) ferment carbohydrates to produce lactate, which helps maintain gut acidity, supports digestion, and prevents pathogenic overgrowth by enhancing immune function and barrier integrity. Bifidobacterium spp. generate lactate from dietary fibers and oligosaccharides, modulating the gut microbiota, improving gut health, and exerting anti-inflammatory effects by promoting the production of beneficial short-chain fatty acids. Faecalibacterium prausnitzii converts carbohydrates into lactate and butyrate, supporting a balanced microbiome and enhancing anti-inflammatory responses, thereby strengthening gut barrier function. *Lactobacillus* plantarum produces lactic acid under anaerobic conditions, promoting gut barrier integrity and potentially aiding in the management of irritable bowel syndrome (IBS) and other inflammatory conditions. Together, these bacteria contribute significantly to gut health by modulating inflammation, supporting digestion, and maintaining a balanced microbiome (Table 4).

## 7 Conclusion and future directions

Lactate, once primarily associated with muscle fatigue and anaerobic metabolism, is increasingly recognized for its complex and dual role in human health. Under normal physiological conditions, lactate serves as a critical energy substrate and plays a pivotal role in metabolic processes like the Cori cycle. However, its excessive accumulation, particularly in diseases like cancer, ischemic stroke, and metabolic disorders, can be detrimental, contributing to disease progression and poor prognosis.

Recent studies have highlighted lactate’s potential as both a boon and a curse. While its role in tumor metabolism, immune regulation, and neuronal signaling shows promise, the harmful effects of chronic lactate accumulation cannot be overlooked. The emerging therapeutic strategies targeting lactate, such as lactate dehydrogenase inhibitors, offer new avenues for disease treatment.

Future research should focus on developing precise methods to regulate lactate metabolism, exploring the role of gut microbiota in lactate production, and understanding the complex interactions between lactate and immune responses. Additionally, investigating the potential of lactate in enhancing exercise performance and as a therapeutic agent in neurological and metabolic diseases warrants attention. Ultimately, advancing our understanding of lactate’s multifaceted role will enable the development of targeted interventions to harness its benefits while mitigating its harmful effects.
